# Investigating the antibacterial effects of some Lactobacillus, Bifidobacterium and acetobacter strains killed by different methods on *Streptococcus mutans* and *Escherichia coli*

**DOI:** 10.22099/mbrc.2019.33582.1399

**Published:** 2019-09

**Authors:** Mohammad Sadegh Safari, Mehrnaz Keyhanfar, Rasoul Shafiei

**Affiliations:** 1 Department of Biotechnology, Faculty of Advanced Sciences and Technologies, University of Isfahan, Isfahan, Iran; 2 Department of Biology, Faculty of Sciences, University of Isfahan, Isfahan, Iran

**Keywords:** Acetobacter, Lactobacillus, Bifidobacterium, killed bacteria

## Abstract

Although there are many health advantages assigned to different live bacteria such as probiotics, some health threatening effects have also been reported. For example, live bacteria can transfer antibiotic resistance genes to other commensal and opportunistic bacteria of gastrointestinal tract. Recently, it was shown that using killed bacteria have some advantages over live ones. In this research, heat, paraformaldehyde and ozone killing methods were used to kill the bacteria. *Acetobacter cerevisiae*, *Lactobacillus acidophilus*,* Bifidobacterium lactis* and traditional vinegar and fermented dairy product (Kumeh) derived bacteria were killed and their antibacterial activity against *Streptococcus mutans *and* Escherichia coli* was investigated. To identify the bacteria isolated from the traditional products, *16S rDNA* gene was partially sequenced. The gene analysis showed vinegar and Kumeh derived bacteria were *Acetobacter* *pasteurianus* and *Lactobacillus crustorum* (LcK) strains respectively. The *S. mutans* growth inhibition was detected in the all concentrations of all killed samples. However, generally, *E. coli* showed more resistant to the killed bacteria than *S. mutans* and the antibacterial effect of heat-killed bacteria against *E. coli* was not observed in the all concentrations for some killed bacteria. Among the pathogenic bacteria, *S. mutans* was the most sensitive one to the killed bacteria with 70% of reduction in its viability. In conclusion, this research showed that different killed bacteria had different effects on other bacteria and the killing method showed an impact on these effects. Overall, paraformaldehyde-killed *L.crustorum* (LcK) showed the best antibacterial activity against *S. mutans*; about 70% decrease in bacterial viability.

## INTRODUCTION

Probiotics are live microorganisms, which when consumed in adequate amounts, confer a health effect on the host [1]. Elie Metchnikoff, a Russian scientist, suggested that lactobacilli might act against the putrefactive effects of gastrointestinal metabolism that contributed to diseases and aging [2]. *Lactobacillus* genus which was studied by Metchnikoff is categorized as probiotics now. Probiotics can confer health effect by adjusting gastrointestinal immune system, implicating such mechanism as production of antibacterial substances like bacteriocins, lactic acid and acetic acid [3,4]. They can also reduce colonization or decrease populations of pathogenic bacteria in the gastrointestinal tract and produce nutrients like vitamin K [5]. *Acetobacter* spp. are a subgroup of acetic acid bacteria which play an important role in food industry specially in vinegar production [6]. Recent studies have shown that these bacteria have some characteristic of probiotics and they are considered as potential probiotics [7]. 

According to the definition of “probiotic” being “alive” is crucial for a microorganism to be considered as probiotic. Some characteristics of probiotics, such as production of antimicrobial substances, effective adherence and colonization in intestinal tract, are related to their viability. Recent researches have shown that killed bacteria can be used instead of live microorganisms due to the risk of live bacteria. The administration of killed bacteria will cause losing some aspect of probiotics but it has some benefits for both probiotic producers and consumers [8]. For example, during storage of fermented probiotic products, special storage conditions are required to control the population of probiotic [9]. Another important issue about administration of live bacteria is probability of antibiotic resistance gene transfer among the gut microbiota [10]. Live microorganisms are able to transfer these genes by mechanisms like conjugation. Penicillin, vancomycin and tetracycline resistance have been identified in *lactobacillus* genus [11]. So, it seems important to avoid health risks by administration of the killed probiotics instead of live ones. 

Different methods are available for killing bacteria. All cellular components can be affected by heat to some degree (outer layers, membrane, enzymes and proteins, DNA, RNA) [12]. Ozone is a powerful oxidizing agent which is known as GRAS (generally recognized as safe) [13]. Ozone has a destructive impact on polyunsaturated fatty acids of cell membrane and some amino acids like tyrosine, tryptophan, phenylalanine, and the sulphur containing amino acids like cysteine and oxidizes them [14,15]. Aldehydes such as paraformaldehyde are mostly used as fixative to stabilize the fine structural details of cells and tissues. The aldehyde group can react with nitrogen and some other atoms of proteins, forming cross link -CH2- called a methylene bridge [16].

In this study, the antibacterial effect of heat killed, ozone killed, and paraformaldehyde killed *A. cerevisiae*, *L. acidophilus*, *B. lactis*, traditional vinegars and fermented dairy (Kumeh) derived bacteria were investigated against *Streptococcus mutans *and* Escherichia coli *by analyzing the growth of the bacteria following treatment with killed bacterial strains. 

## MATERIALS AND METHODS

**Bacterial strains and growth conditions: **In the current study, antibacterial effects of two diverse probiotic strains, *L. acidophilus *(ATCC 4356) and *B. animalis *subsp. *lactis* (DSM 10140) (both provided by the Department of Microbial Biotechnology, University of Isfahan, Iran), *A. cerevisiae*??*A. cerevisiae* was grown in modified Carr selective medium (yeast extract 0.5%, peptone 1%, glucose 2%, absolute ethanol 3%, acetic acid 0.5%, ammonium phosphate 0.01%, magnesium sulfate 0.01%, and agar 1.5%) which is an specific isolation medium for *acetobacter* strains [17]. For liquid culture, one colony was taken from Carr agar plate and grown 24 h in 50 ml of Carr broth at 30 C. Three bacterial strains were isolated from Kumeh and two types of traditional vinegar (mulberry and grapes) using selective culture medium. The MRS broth medium was used to isolate bacteria from Kumeh and modified Carr broth medium to isolate bacteria from vinegars. The strains were cultivated on isolation media for 7 days followed by aseptically transferring 5 random colonies to solid medium to grow for 24 hours. The incubation temperature was 30 C for all bacteria except for Kumeh isolated bacteria, incubated at 37 C. Morphological characteristics of selected colonies from each plate were checked out using Gram staining and catalase tests. Further experiments for identification and characterization by *16s rDNA* gene amplification of the isolated bacteria were carried out on just one of the colonies. Having prepared the growth rate curves of the bacteria (data are not shown), the bacteria were harvested in the exponential phase accordingly resuspended in PBS and centrifuged twice at 1800×*g *(Pars Azma Centrifuge, Iran) for 10 min. Bacterial concentration in PBS was obtained using a Thoma cell counting chamber and required concentrations (10^7^ cell/ml, 10^6^ cell/ml, 10^5 ^cell/ml) were prepared by diluting the main bacterial stocks.

**Killing the bacteria: **? ? ??*Acetobacter* strains) to confirm the effectiveness of the killing method.

**DNA extraction: **The bacterial cells were grown overnight (as mentioned above) and chromosomal DNA was isolated as described previously [18]. The aqueous phase contained purified DNA and was stored at -20C and then was directly used for subsequent experiments.

**PCR analysis of** ***16S rDNA*** **: **PCR amplification of the partial *16S rDNA* gene of the isolated bacterial strains from Kumeh and two types of traditional vinegars were performed using the following universal forward and reverse primers: 27F: 5’AGA GTT TGA TCC TGG CTC AC3’ [19] and 1492R: 5’CGG TTA CCT TGT TAC GAC TT3’ [20?????^21^]. The 1200 bp sequences were PCR amplified and sequenced by Gen Fanavaran Ltd. All the sequences were analyzed by CLC Main workbench 5 program and were searched for homologies in the BLASTN database (National Center for biotechnology Information) using the basix local alignment search tool.

**Phylogenetic Analysis: **After sequencing the amplicons, to compare the similarities among the obtained sequences the phylogenetic trees were constructed by the neighbor-joining statistical method using Mega version 6. The sequence data were sampled 1,000 times for bootstrap analysis with 50% cut-off. *Gluconobacter oxydans* strain DSM 3503 and *Bacillus subtilis* subsp. *spizizenii* strain NBRC 101239, were used as outgroups.

**Antibacterial activity assay: **Screening of all killed bacteria for their qualitative antibacterial activity was performed by employing microdilution method [22]. One ml cultures of *S. mutans* (ATCC 35668) and non-pathogenic *E. coli* (ATCC 25922) ?^23^] to yield a density of bacterial cells about 1.5×10^7^ cells/ml (0.05 McFarland). 50 μl of the bacterial suspension was added to each well of a 96 wells plate. Then, 100μl of cation adjusted Muller Hinton Broth and 100 μl of each killed bacterial samples with ratio of 2:5 were added to each well. One well was kept as positive control, contained broth, inoculum and tetracycline (1 mg/ml), and one well as negative control, contained broth and inoculum and one well (just broth) to make sure there is no contamination in the broth. The plates were covered and incubated overnight at 37?. To indicate the bacterial growth inhibition, optical density of the wells were measured by multiwell scanning spectrophotometer (ELISA reader) at 580nm. 

**Lactic acid analysis by Gas Chromatography: **Supernatant of Lck bacteria was analyzed to assure that bacterial sample does not contain lactic acid. The paraformaldehyde-killed LcK sample soluble was centrifuged at 8000×g for 2 min. The supernatant was collected and then filtrated by 0.22 μm pore size membrane filter. One μl of the filtrate was injected to GC (Aglinet, model 6890N) equipped with INNOWAX column and the concentration of lactic acid was assessed.

**Statistical Analyze: **The results for inhibition tests were expressed as the mean±SD of three independent experiments. The data were analyzed by one-way ANOVA test with IBM SPSS Statistics. A P<0.05 was considered to represent a statistically significant difference between compared data sets.

## RESULTS

**Figure 1:**
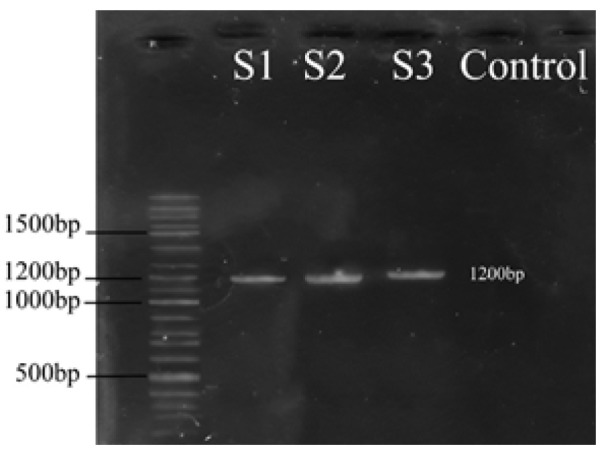
Gel electrophoresis of PCR for Lck (S1), ApG (S2), and ApM (S3)

**Figure 2:**
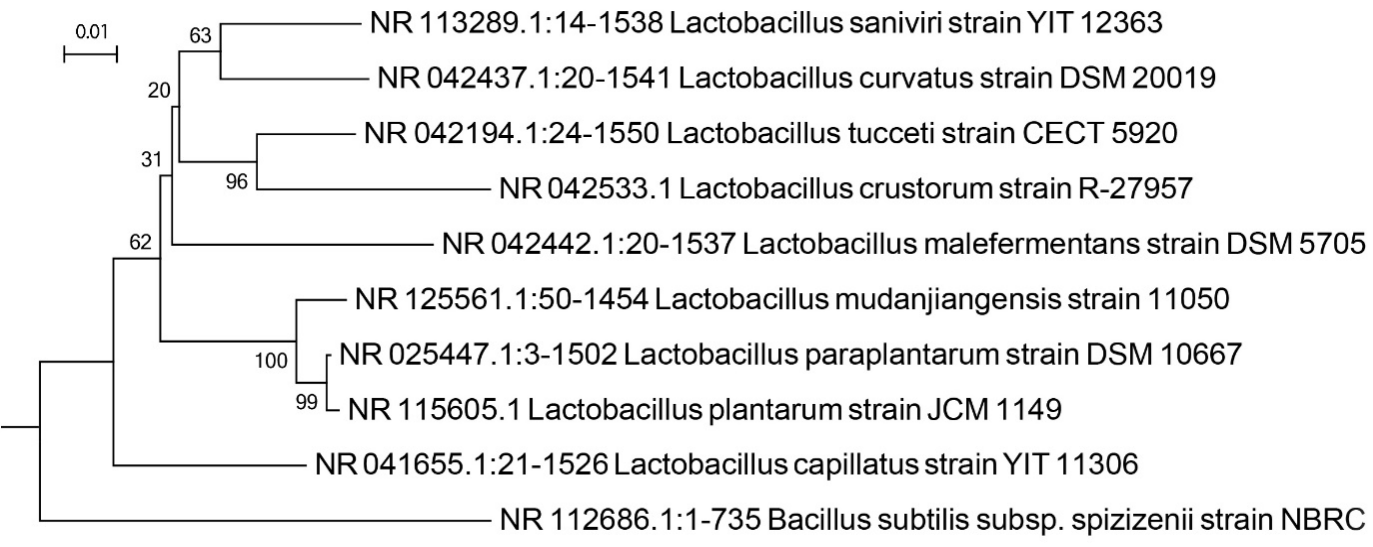
Phylogenetic tree based on *16S rDNA* sequence analysis, showing the phylogenetic placement of strain isolated from komeh (LcK). The percentage of replicate trees in which the associated taxa clustered together in the bootstrap test (1000 replicates) are shown next to the branches. The tree was rooted on *Bacillus subtilis*

The isolated bacterium from Kumeh was rod-shaped, Gram-positive, and catalase negative with circular, smooth, and white colonies on MRS agar. In figure 1, S1 shows PCR of amplicon for Kumeh derived bacteria (molecular size of 1200 bp) was associated with the calculated size of a target gene and shows the single band of PCR. Table 1 (in supplement file) presents the DNA sequences target genes presents. Partial sequencing of *16S rDNA* indicated 99% identity with *L. crustorum*. Therefore the isolated bacterium was confirmed as *L. crustorum* and abbreviated as LcK in this study. The phylogenetic placement of strain isolated from Komeh is shown in Figure 2. 

**Figure 3:**
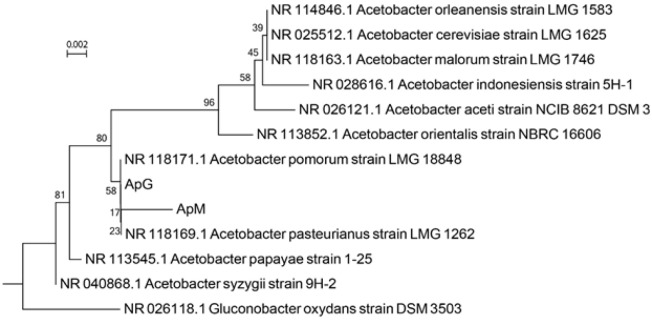
Phylogenetic tree based on *16S rDNA* sequence analysis, showing the phylogenetic placement of strains isolated from mulberry (ApM) and grape vinegar (ApG). The percentage of replicate trees in which the associated taxa clustered together in the bootstrap test (1000 replicates) are shown next to the branches. the tree was rooted on *Gluconobacter oxydans*

The isolated bacteria from mulberry and grapes vinegars were Gram negative coccobacilli and catalase positive with circular, rough, and white colonies on MRS agar. In figure 1, S2 and S3 show PCR of amplicon for mulberry and grapes vinegars derived bacteria ( molecular size of 1200 bp) was associated with the calculated size of a target gene and shows the single band of PCR. Partial sequencing of *16S rDNA* of both isolated bacteria indicated 99% identity with *Acetobacter pasteurianus *species. Therefore the isolated bacterium were confirmed as strains of *A. pasteurianus* and abbreviated as ApM (Mulberry vinegar isolated strain) and ApG (Grape vinegar isolated strain) in this paper. The phylogenetic placement of strains isolated from mulberry and grape vinegar is shown in Figure 3.

**Figure 4:**
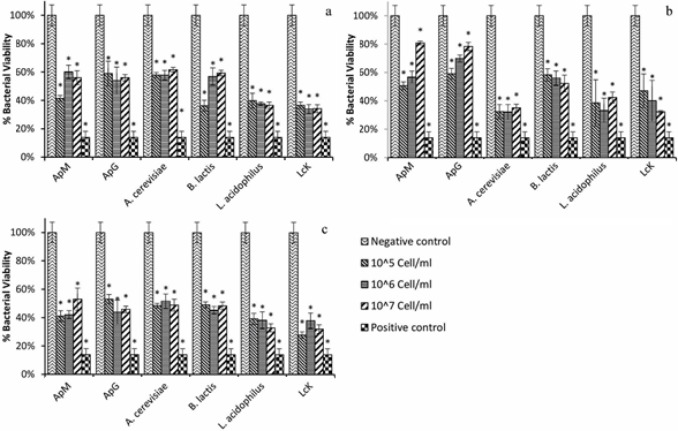
Antibacterial effect of different concentrations of **a**) heat-killed, **b)** Ozone killed, and **c**) paraformaldehyde killed bacteria on *S.* *mutans* ATCC35668. The *S.* *mutans *bacteria were grown in 96 well plates, and were cultured in absence (Negative control) or presence of killed bacteria. These results were obtained as described above, and the relative proportion % of untreated (negative control) bacteria at 24 h. Bars, means of triplicates±SD.*P<0.05, as compared with the relative growth of control sample

Antibacterial activity of the killed bacteria were examined using two different bacterial pathogens, including a Gram-positive strain, *S. mutans* and a Gram-negative bacteria strain, *E.coli*. The growth inhibition was verified after 24 h of incubation and as shown in Figure 4a, all of the heat-killed bacteria had antibacterial and growth inhibition activity but among them, Lck expressed the best results on *S. mutans*; about 65% (*P*<0.05) decrease in bacterial growth in each examined concentrations. As it is shown in Figure 4a, there was no significant difference between concentrations’ growth inhibition activity in each killed sample and the effect was not dependent to the concentration of killed bacteria (Ozone-killed, Heat-killed, and Paraformaldehyde-killed). Three samples of ozone killed bacteria, *A. cerevisiae*, *L. acidophilus *and Lck, had almost similar effect on *S. mutans* (Fig. 4b). A 60% decrease in bacterial growth was seen as a result of applying these samples. All the paraformaldehyde killed bacteria also revealed great significant impact on bacterial growth of *S. mutans*. Similar to heat killed bacteria, Lck expressed the best antibacterial effect and caused 70% decrease in bacterial growth (Fig. 4c).

**Figure 5:**
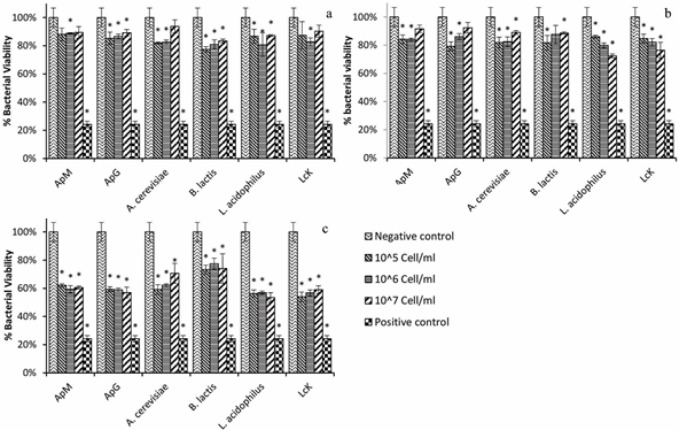
Antibacterial effect of different concentrations of **a)** heat-killed, **b)** ozone killed, and **c)** paraformaldehyde killed on *E. coli *ATCC25922. The *E. coli *bacteria were grown in 96 well plates, and were cultured in absence (Negative control) or presence of killed bacteria. These results were obtained as described above, and the relative proportion % of untreated (negative control) bacteria at 24 h. Bars, means of triplicates±SD. *P<0.05, as compared with the relative growth of control sample

In contrast to the effect of killed bacteria on *S. mutans* which was able to be detected in all concentrations of all samples, antibacterial effect of heat-killed bacteria against *E. coli* was not observed in some situations. Generally, *E. coli* showed more resistant to the killed bacteria than *S*.*mutans*. The best antibacterial activity of the heat-killed bacteria was observed in *B.lactis* which decreased bacterial growth about 20% (Fig. 5a). Ozone killed bacteria showed similar effect on *E. coli* and again the best result was achieved by *B. lactis* with 30% decrease in bacterial growth (Fig. 5b). The paraformaldehyde killed bacteria had greater impact on *E.coli *viability in comparison with other methods. All the killed bacteria decreased *E.coli* growth about 40% (Fig. 5c).

## DISCUSSION

Fermented foods such as dairy products and vinegar have been recognized as valuable products to human health. Many species and strains of lactic acid bacteria and acetic acid bacteria were isolated and identified from these products. Partial sequencing of *16S rDNA* gene of isolated bacteria confirmed that Kumeh isolated bacteria as *L.crustorum* and vinegar isolated bacteria as *A. pasteurianus*. Although these bacteria have shown some aspects of probiotics, they are not well known as probiotics and further studies are required [7].

The *S. mutans* is considered to play an important role in dental caries [24]. Probiotic containing products are known to contain compounds that reduce the risk of dental caries [25]. Antibacterial effects of different probiotics on *Streptococcus* species were investigated in other studies. For instance, *B. animalis* subsp. *Lactis* DN-173010 significantly reduced the salivary levels of *S. mutans* in orthodontic patients [26]. A statistically significant reduction of the *S. mutans* levels was observed after consumption of the bacteria via the straw and the tablets [27]. The effect of *Lactobacillus* GG on *S. sobrinus* and their experiments showed a 10-fold inhibition in growth of *S. sobrinus* by Lactobacillus GG substances [28]. *Lactobacillus* and *Bifidobacterium* species had moderate antibacterial activity, a growth inhibition from 20% to 70% (Fig. 3). In addition, Lck expressed the best antibacterial effect and caused 70% decrease in *S. mutans* viability (Fig. 3c).

Recent researches described a new enzymatic functionality for the S-layer (surface layer) of *L. acidophilus *ATCC 4356. The S-layer showed an endopeptidase activity against the cell wall of *S.* *enterica* [29]. Moreover, S-layer acted synergistically in combination with nisin to inhibit the growth of *S. enterica*, *Staphylococcus aureus* and *Bacillus cereus*. [30]. The growth of isolated mutans streptococci strains was also inhibited by *Lactobacillus* strains at bacterial concentration of ≥10^7 ^CFU/ml [28]. Schwendicke et al. assessed antibacterial activity of viable and heat-inactivated *B. animalis* BB12 *in vitro*. Viable *B. animalis* did not significantly reduce cariogenicity of *S. mutans*, whilst heat-inactivated *B. animalis* decreased cariogenicity of *S. mutans* in dentinal cavities [31]. Our data imply that all of the killed bacteria with different methods expressed antibacterial activity against *S. mutans*. However, a better antibacterial activity was shown when bacteria were killed with paraformaldehyde in comparison with heat killed and ozone killed bacteria.

Coconnier et al investigated the antibacterial effect of the adhering human *L. acidophilus* strain LB against a wide range of gram-negative and gram-positive pathogens and showed *L. acidophilus* decreased the viability of *E. coli, S. typhimurium, S. flexneri, B. cereus, P. aeruginosa*, and *Enterobacter* spp. without any significant inhibition of other lactobacilli and bifidobacteria [32]. Inoculation of yoghurt and soymilk sample containing viable *B. lactis *(Bb-12) and *B. longum *(Bb-46) with *E.coli* or *S. aureus* showed more than 90% decrease in the count of *E. coli* [33]. In the current study, most of the killed bacteria had antibacterial effect on *E. coli*. There is also significant difference between killing methods. Paraformaldehyde killed bacterial strains showed more antibacterial activity in comparison with other methods which was up to 40% loss of growth. It is probably because paraformaldehyde has less damage on bacterial proteins and other biological structures. 

Viable probiotics may suppress the proliferation of pathogens by different methods. They can produce diverse antimicrobial metabolites including bacteriocins, lactic acid and acetic acid [34]. In this study, killed bacteria were used to assay their antibacterial activity. According to GC analysis, there was no lactic acid present in the inoculum, so the antibacterial effect cannot be admitted to these metabolites. To the best of our knowledge, there is no evidence that shows acetobacters can produce bacteriocins, so the antibacterial activity of killed bacteria is probably related to the killed bacterial extract structures such as lipopolysaccharide, inner and outer membrane proteins, peptidoglycan and etc. In conclusion, in this research it was shown that the studied killed bacteria had different effects on other bacteria -mostly growth inhibitory effect- and the method of killing had an impact on these effects. Although the mechanism(s) by which the selected probiotics and acetic acid bacteria reduced the viability of pathogenic bacteria remains to be elucidated, this study indicate that killed bacteria components could be an important factor which has some impacts on the homeostasis of the flora in the gastrointestinal tract. Further studies are needed to clarify the mechanism(s) of this effect.

## Supplementary Materials

Supplement
