# Supplementary material for: Investigating the antibacterial effects of some Lactobacillus, Bifidobacterium and acetobacter strains killed by different methods on Streptococcus mutans and Escherichia coli
Source: Mol Biol Res Commun. 2019 Sep;8(3):103–11. doi: 10.22099/mbrc.2019.33582.1399 (PMC6802690; doi:10.22099/mbrc.2019.33582.1399)
Supplement: Supplement [file mbrc-8-103.s001.pdf]

Table 1: Partial DNA sequence of *16S rDNA* of isolated strains

Isolated Strain	Partial *16s rDNA* gene sequence
**LcK**	GCTCCCGATAAAGCTTAAGCTTTAAAATACCAATTAACGTTTCGCTTCCAAAAGTTAAGGCTCGTACACCAAATTAAGC<br>TCGTTGCGCTTCTTGGAATGGTCCAGAACTGTCATGTACTTCTCGATTCTCTACTCACAAAGGGAATCCCCTGTACCTAG<br>TCCACCACGTACCACAGCAGTTGAGCACAACACTCTACAACCCAATTCAAGGCGTTGCTTGCGTTGGGAAAAATAGTC<br>ACGGTCGTAAGTCCCCCCGCGGAGACATCTCTCTGGCCGCCGTTGTGCCTCCTTCCTCCCCCCCTGCAGCTGAGCAGTAG<br>AGGAATACTGCAGCCCCTGAGGGGACCACGTTGTCAGCGACGCTGTCCCCCTCTTATCCGCTCCCATTGGTTTAGTAGA<br>ATTGGCTACAGTCTAGCCTATCATGCGACGTGTAGCGCACGTGCTTCTCCCTTAGCGATCACTAGCGCCTAGGGTAATTTTGCCCCCACCCCGGTAAAAGG<br>AGGGGGAAAACTTTTCTGATTTATGGGGGAAAAAGATGTTGGGGGTTTTTACGTTGAAGTGAAACCCTCGGTCTACCGAGAAATCTTAGAAATCTGTAAA<br>CTGAGTGCAGAGAGGGAGGGGAATTCACAGTAGGGGGGAATTGGAAAATATGGAAAACCCCGGGGGGAAGGGGGTCTCCGGTCGTATTGACCTGAGAG<br>TCGAAAACATGGGAGCAAACAGGATAGATACCCTGGTAGTCCCGCGGTAAACGAGGAGTGTTAAGATTGGGGGGTTTAGCCCTTCAGTGTCGCAGTTAA<br>CGCATTAAGCACTCCGCCTGGGGAGTACGATCGCAAGATTGAAACTCAAAGGAATTGACGGGGGCCCGCACAAGCGGTGGAGCATGTGGTTTAATTCGA<br>AGCAACGCGAAGAACCTTACCAGGTCTTGACAGTACATGAAGAGCTAAGGATGAGTGTTTCCCTTAGGGGACATGGATCCAGGTGGTGCATGGTGTCGTC<br>AACTCGTGTTGTGAGATGTTGGGTTAAGTTCCGCAACGAACGCAACCCTTTTTATCAGTTGCCAGCATTCAGGGGGGCGCCTCTGTAGAGAGACCGGCGG<br>CAACACGGAGGAAGGAGGGGGGGACGTCGACTCGTCATCATCCTTATGACGTCGGGGACTCCCCTGGTGCAACAGTCGCTGCGACAGGGGGAGAATAGG<br>CGAGGGTAACCAAATCATCTATAACCGATGTCAGATCGGATAGTACGCTGCACATCGCGTGCACGAAGAGGGAATCGCTAGTGATCGCGGATCAGCACA<br>CCGCGGGGAATACGTTCCGGGCCCGTGCACACCGCCCGTCACACCACGAGAGTTTGATCGCCCAAAGTCGGGTAGAGACCCTCAGGGTGCAGTTACTCAG<br>TTTATTTTCCTCTCAGCGGA
**ApM**	CCCGCTCGCTGACGACGGTGGTCGGGCTGCGGTCCTTGCGGTTCGCTCCCGGCTTAAGGTGTGCTAATACCGCATGACACCTGAGGGTCAAAGGCGCAAG<br>TCGCCTGTGGAGGAGCCTGCGTTTGATTAGCTAGTTGGTGGGGTAAAGGCCTACCAAGGCGATGATCAATAGCTGGTTTGAGAGGATGATCAGCCACACT<br>GGGACTGAGACACGGCCCAGACTCCTACGGGAGGCAGCAGTGGGGAATATTGGACAATGGGGGCAACCCTGATCCAGCAATGCCGCGTGTGTGAAGAAG<br>GTCTTCGGATTGTAAAGCACTTTCGACGGGGACGATAATGACGGTACCCGTAGAAAAAGCCCCGGCTAACTTCGTGCCAGCAGCCGCGGTAATACGAAGG<br>GGGCTAGCGTTGCTCGGAATGACTGGGCGTAAAGGGCGTGTAGGCGGTTTGTACTGACGACAGCCATGCAGCACCTGTGTTAAGGTCCCTTGCGGGAAAA<br>AACATCTCTGCTTGCAGCCTCTACATTCAAACCCTGGTAAGGTTCTGCGCGTTGCTTCGAATTAAACCACATGCTCCACCGCTTGTGCGGGCCCCCGTCAAT<br>TCCTTTGAGTTTCAACCTTGCGGCCGTACTCCCCAGGCGGTGTGCTTAACGCGTTAACTGCGACACTGAATGACTAAGTCACCCAACATCTATCACACATC<br>GTTTACAGCGTGGACTACCCGGGATCTAATCCTGTTTGCTCCCCCGCTTTCGCGCCTCACCGTCAGTAATGAGCCACGTTGCCGCCTTCCCCCCGGGGTTCT<br>TCCCATATCTACAAATTCACCTCTAACTGGGAATTCACAACCTCTCTCACACTCTATCTGCCGTATCAATGCACTCCCCGTTAACCCGGGATTCACTCTGAT<br>GTACAAACGCCACACGCCCTTACGCCAGTCATTCCAGAACGCTTTCCCCTTTTATTACGCGGCTGTGGGACAAATTATCCGGGCTCTTCTACGGTACGGCA<br>TCTCGTCCCGTCGAAATGCTTAAATCCAAAACTTCTCACACCGGCATGTGGATCAGGTGCCCCATGTCAAATTCCCCTGTTCTCCCTAAGAATTGGGCCGG<br>TTCATCCCAGGGGGTGATATCCTTCAACCCCTTTGAAATCCCTGGGAGGCTTACCCCCAATATTATTAACCAGGTCCCCCAGGGAATTGGCTTTAACTCAG<br>GGTTTGGGGTTAAACCCCTTCCCGGGTTCCCCCCCGGAAAATCCACCGTTTCCCCCCCCAAAGCAAACTTGGGATTAGTGAATGCCTGGGGGCAGTTTCAA<br>AAAAAAAAAAAAAAAAAAAAAAATTTTTTACTTTATTTCTCCCCCCCCCCCCCCAACCCCCCCCCCCC
**ApA**	ATATATAGGCGAAAGGTGTTAAGAAAGTCTGCGGTATATAGCGCGTGTTATGGGCCAGTGGCGGTTTTGGGTCTAAAAGCGGGGGGGGGGGGGGGTTTTT<br>TTGTTTCACTTTAGGGAACCGTAACGCGTAGGTATCTATCCATGGGTGGGGGATAACACTGGGAAACTGGTGCTAATACCGCATGACACCTGAGGGTCAA<br>AGGCGCAAGTCGCCTGTGGAGGAGCCTGCGTTTGATTAGCTAGTTGGTGGGGTAAAGGCCTACCAAGGCGATGATCAATAGCTGGTTTGAGAGGATGATC<br>AGCCACACTGGGACTGAGACACGGCCCAGACTCCTACGGGAGGCAGCAGTGGGGAATATTGGACAATGGGGGCAACCCTGATCCAGCAATGCCGCGTGT<br>GTGAAGAAGGTCTTCGGATTGTAAAGCACTTTCGACGGGGACGATGATGACGGTACCCGTAGAAGAAGCCCCGGCTAACTTCGTGCCAGCAGCCGCGGTA<br>ATACGAAGGGGGCTAGCGTTGCTCGGAATGACTGGGCGTAAAGGGCGTGTAGGCGGAAGAAGCCCCGGCTAACTTCGTGCCAGCAGCCGCGGTAATACG<br>AAGGGGGCTAGCGTTGCTCGGAATGACTGGGCGTAAAGGGCGTGTAGGCGGTTTGTACAGTCAGATGTGAAATCCCCGGGCTTAACCTGGGAGCTGCATT<br>TGATACGTGCAGACTAGAGTGTGAGAGAGGGTTGTGGAATTCCCAGTGTAGAGGTGAAATTCGTAGATATTGGGAAGAACACCGGTGGCGAAGGCGGCA<br>ACCTGGCTCATTACTGACGCTGAGGCGCGAAAGCGTGGGGAGCAAACAGGATTAGATACCCTGGTAGTCCACGCTGTAAACGATGTGTGCTAGATGTTGG<br>GTGACTTAGTCATTCAGTGTCGCAGTTAACGCGTTAAGCACACCGCCTGGGGAGTACGGCCGCAAGGTTGAAACTCAAAGGAATTGACGGGGGCCCGCAC<br>AAGCGGTGGAGCATGTGGTTTAATTCGAAGCAACGCGCAGAACCTTACCAGGGTTTGAATGTAGAGGCTGCAAGCAGAGATGTTTGTTTCCCGCAAGGGA<br>CCTCTAACACAGGTGCTGCATGGCTGTCGTCAGCTCGTGTCGTGAGATGTTGGGTTAAGTCCCGCAACGAGCGCAACCCCTATCTTTAGTTGCCATCAGGT<br>TGGGCTGGGCACTCTAGAGAGACTGCCGGTGACAAGCCGGAGGAAGGTGGGGATGACGTCAAGTCCTCATGGCCCTTATGTCCTGGGCTACACACGTGCT<br>ACAATGGCGGTGACAGTGGGAAGCTAGGTGGTGACACCATGCTGATCTCTAAAAGCCGTCTCAGTTCGGATTGCACTCTGCAACTCGAGTGCATGAAGGT<br>GGAATCGCTAGTAATCGCGGATCAGCATGCCGCGGTGAATACGTTCCCGGGCCTTGTACACACCGCCCGTCACACCATGGGAGTTGGTTTGACCTTAAGC<br>CGGTGAGCGAACCGCAAGGACGCAGCCGACTCATCGTCAGTCCTGCCACTGT
